# Short-Term Morbidity and Mortality in Patients Undergoing Radical Cystectomy at a Tertiary Care Center: A Prospective Observational Study

**DOI:** 10.7759/cureus.95183

**Published:** 2025-10-22

**Authors:** Saundarya Kumar Verma, Saleem Wani, Abdul Rouf Khawaja, Sajad A Malik, Sajjad A Para, Arif Hamid, Saqib Mehdi, Mudasir Ahmad Tantray, Tufeel Ahmad Khan

**Affiliations:** 1 Department of Urology, Sher-i-Kashmir Institute of Medical Sciences (SKIMS), Srinagar, IND

**Keywords:** clavien-dindo classification, ileal conduit, muscle-invasive bladder cancer, radical cystectomy, urinary diversion

## Abstract

Background: Radical cystectomy (RC) is the standard treatment for muscle-invasive bladder cancer (MIBC) but is associated with significant perioperative morbidity and mortality. This study evaluates short-term (90-day) morbidity and mortality outcomes following RC at Sher-I-Kashmir Institute of Medical Sciences (SKIMS), Srinagar, to identify complications and risk factors.

Methods: This prospective observational study, conducted from February 2023 to July 2025, included 173 patients undergoing RC for MIBC or high-risk non-MIBC (NMIBC) at SKIMS. Patients underwent RC with ileal conduit (IC) or orthotopic neobladder (NB) diversion. Complications within 90 days were graded using the Clavien-Dindo classification. Data on preoperative, operative, and postoperative variables were analyzed using IBM SPSS Statistics for Windows, version 24, with associations tested at a 5% significance level (P < 0.05).

Results: Of 173 patients (86.7% male, mean age 59.9 years), 52.6% (91/173) experienced complications, with 15.6% (27/173) classified as major (Grades 3-5). The most frequent complications were superficial wound infections (9.8%), postoperative blood transfusions (8.7%), and subacute intestinal obstruction (5.8%). The 90-day mortality rate was 3.4% (6/173), primarily due to septic shock and cardiovascular events. Predictors of complications included diabetes mellitus, advanced disease stage, higher American Society of Anesthesiologists (ASA) score, low preoperative hemoglobin, low serum albumin, longer operative time, and increased blood loss. Orthotopic NB patients had longer operative times and hospital stays but fewer complications due to selective patient criteria.

Conclusion: RC at SKIMS carries a 52.6% complication rate and 3.4% mortality rate within 90 days. Optimizing patient selection, surgical techniques, and perioperative care is critical to reducing morbidity and improving outcomes in this high-risk procedure.

## Introduction

Bladder cancer is a major global health concern, ranking as the fourth most common cancer among men in the USA [1], with a median diagnosis age of 72 years and a threefold higher incidence in men. Radical cystectomy (RC) is the standard treatment for muscle-invasive bladder cancer (MIBC) and select high-risk non-MIBC (NMIBC), yet it is associated with high morbidity (30-77%) and mortality (0.8-8%) within 90 days [2,3]. Despite advancements in surgical techniques, anesthesia, and postoperative care, the mean hospital length of stay (LOS) remains nine to 11 days, with 58-77% of patients experiencing complications within 30 days and a 27% readmission rate. Complications such as infections, gastrointestinal issues, and bleeding increase hospital stays and costs [4]. Perioperative mortality is a concern, particularly for elderly patients or those with comorbidities, underscoring the need to address surgical morbidity for treatment planning, clinical trial design, new technique evaluation, and patient education.

Transurethral resection of bladder tumors is the gold standard for diagnosing MIBC, which accounts for 20-30% of initial presentations, with 20% of non-muscle-invasive cases progressing to this stage. Urothelial carcinoma dominates, comprising over 90% of bladder tumors, with squamous cell carcinoma, adenocarcinoma, and neuroendocrine tumors being less common. Despite aggressive multimodal therapy involving surgery, chemotherapy, and radiation, recurrence is frequent, and untreated muscle-invasive cases face an 85% mortality rate within two years. Postoperative mortality ranges from 0.8% to 8%.

Risk factors for complications include preoperative factors (e.g., age, comorbidities, cancer stage), operative factors (e.g., surgical duration, blood loss), and postoperative factors (e.g., ICU stay, stent removal). Complications increase LOS, care costs, and in-hospital mortality risk, making their prediction and prevention critical. Hospital readmission after RC, affecting 20-30% of patients within 30 days, remains a significant challenge, unchanged for decades, and is a key driver of healthcare costs. This study addresses the limited data on readmission incidence and risk factors post-RC, aiming to identify preventable causes to improve patient outcomes.

## Materials and methods

This prospective observational study was conducted at the Department of Urology, Sher-i-Kashmir Institute of Medical Sciences (SKIMS), Srinagar, from February 2023 to July 2025. A total of 173 patients undergoing RC for bladder cancer were enrolled after obtaining ethical approval and informed consent. Inclusion criteria included patients with MIBC or high-risk NMIBC undergoing RC with ileal conduit (IC) or orthotopic neobladder (NB) diversion. Exclusion criteria were cystectomy for non-bladder cancer indications or refusal to participate.

Preoperative assessment

Patients underwent detailed evaluations, including demographics, comorbidities, American Society of Anesthesiologists (ASA) score, smoking history, operative (surgery duration, blood loss), and postoperative (length of stay, ICU care, reoperations).

Surgical procedure

RC was performed with either IC or orthotopic NB diversion. Complications within 90 days were recorded and graded using the Clavien-Dindo classification and categorized into 11 types (e.g., gastrointestinal, infectious, wound-related).

Statistical analysis

Data were analyzed using IBM SPSS Statistics for Windows, Version 24.0 (released 2016, IBM Corp., Armonk, NY). Categorical variables were expressed as frequencies and percentages, with associations tested using t-tests, chi-square tests, or ANOVA at a 5% significance level (P < 0.05). The sample size (n = 173) was calculated for 95% confidence and 90% power, assuming a 5% complication proportion and 4% error margin.

Primary and secondary outcomes

The primary outcomes were the rates of morbidity (defined as any postoperative complication within 90 days, graded using the Clavien-Dindo classification system) and mortality (90-day mortality rate) following RC. Secondary outcomes included the associations between preoperative (e.g., age, gender, comorbidities, ASA score, hydronephrosis, neoadjuvant chemotherapy (NAC)), operative (e.g., operative time, blood loss), and postoperative factors (e.g., length of hospital stay) and the primary outcomes, as well as comparisons between urinary diversion types (IC vs. orthotopic NB).

## Results

Of 173 patients, 86.7% were male, with a mean age of 59.9 years (median 65.5 years). Most (77.5%) had an ASA score of 2, and 74% had comorbidities, primarily hypertension (58.4%) and diabetes (42.8%). Smoking was reported in 53.2% of patients. Muscle-invasive disease was present in 86.1%, and 40% had preoperative hydronephrosis, significantly associated with ileal conduit diversion (χ² = 5.484, P = 0.019). NAC was used in 14% of patients, all in the conduit group (Table 1, Table 2).

**Table 1:** Patient demographics and clinical characteristics (n = 173)

Characteristic	Overall (n = 173)	Conduit (n = 155)	Neobladder (n = 18)	Test statistic	P-value
Male, n (%)	150 (86.7%)	136 (87.7%)	14 (77.8%)	χ² = 1.418	0.234
Mean age (years)	59.9 ± 10.2	60.2 ± 10.3	57.1 ± 9.8	t = 1.324	0.187
ASA score 2, n (%)	134 (77.5%)	122 (78.7%)	12 (66.7%)	χ² = 1.496	0.222
Comorbidities, n (%)	128 (74%)	118 (76.1%)	10 (55.6%)	χ² = 3.771	0.052
Smoking, n (%)	92 (53.2%)	85 (54.8%)	7 (38.9%)	χ² = 1.678	0.194
Muscle-invasive, n (%)	149 (86.1%)	136 (87.7%)	13 (72.2%)	χ² = 3.347	0.067
Hydronephrosis, n (%)	64 (40%)	62 (40%)	2 (1%)	χ² = 5.484	0.019
Neoadjuvant chemotherapy, n (%)	24 (14%)	24 (15.5%)	0 (0%)	χ² = 3.187	0.075

**Table 2:** Operative and postoperative outcomes

Parameter	Overall (n = 173)	Conduit (n = 155)	Neobladder (n = 18)	Test statistic	P-value
Mean operative time (min)	293.6 ± 45.2	290.6 ± 44.8	319.4 ± 48.7	t = -2.318	0.022
Mean blood loss (mL)	977.5 ± 346.5	986.7 ± 353.7	1050.2 ± 488.4	t = -0.529	0.598
Mean hospital stay (days)	10.26 ± 3.33	10 ± 3.33	16.25 ± 5.3	t = -6.058	<0.001

Complications

Complications occurred in 91 patients (52.6%), with 27 (15.6%) experiencing major complications (Grades 3-5). The 90-day mortality rate was 3.4% (6/173), primarily due to septic shock (four cases) and cardiovascular events (two cases) (Table 3).

**Table 3:** Spectrum of postoperative complications (Clavien-Dindo classification)

Complication	Overall (n = 173)	Conduit (n = 155)	Neobladder (n = 18)
				Grade 1	37 (21.4%)	28 (18.1%)	9 (50%)
Superficial wound infection	17 (9.8%)	13 (8.4%)	4 (22.2%)				
				Pulmonary (atelectasis/consolidation)	8 (4.6%)	6 (3.9%)	2 (11.1%)
Urinary incontinence	1 (0.6%)	0 (0%)	1 (5.6%)				
				Pleural effusion	2 (1.2%)	2 (1.3%)	0 (0%)
Lymphorrhea	9 (5.2%)	7 (4.5%)	2 (11.1%)				
				Grade 2	27 (15.6%)	21 (13.5%)	6 (33.3%)
Blood transfusion/TPN	15 (8.7%)	11 (7.1%)	4 (22.2%)				
				Subacute intestinal obstruction	10 (5.8%)	8 (5.2%)	2 (11.1%)
Deep vein thrombosis	2 (1.2%)	2 (1.3%)	0 (0%)				
				Grade 3a	13 (7.5%)	12 (7.7%)	1 (5.6%)
Wound dehiscence	9 (5.2%)	8 (5.2%)	1 (5.6%)				
				Anastomotic leak (ureteroileal)	2 (1.2%)	2 (1.3%)	0 (0%)
Omental hernia	1 (0.6%)	1 (0.6%)	0 (0%)				
				Post-op fever with collection	1 (0.6%)	1 (0.6%)	0 (0%)
Grade 3b	5 (2.9%)	5 (3.2%)	0 (0%)				
				Intestinal obstruction	2 (1.2%)	2 (1.3%)	0 (0%)
Anastomotic stricture	1 (0.6%)	1 (0.6%)	0(0%)				
				Rectal injury	2 (1.2%)	2 (1.3%)	0(0%)
Grade 4	3 (1.7%)	3 (1.9%)	0 (0%)				
				Enterocutaneous fistula	1 (0.6%)	1 (0.6%)	0 (0%)
Sepsis	2 (1.2%)	2 (1.2%)	0 (0%)				
				Grade 5 (mortality)	6 (3.4%)	6 (3.9%)	0 (0%)

Pathological findings

Urothelial carcinoma was predominant (93.6%), with no significant difference in histological types between diversion groups (χ² = 0.135, P = 0.714). NB patients were more likely to have organ-confined disease (77.8% vs. 24.5%; χ² = 19.073, P < 0.001) (Table 4).

**Table 4:** Pathological staging (pTNM)

Staging	Overall (n = 173)	Conduit (n = 155)	Neobladder (n = 18)	Test statistic	P-value
Organ-confined (pN0 or ≤pT2)	52 (30%)	38 (24.5%)	14 (77.8%)	χ² = 19.073	<0.001
Extravesical (≥pT3 or ≥pN1)	121 (70%)	117 (75.5%)	4 (22.2%)	χ² = 12.073	<0.001

## Discussion

RC remains the cornerstone of curative treatment for MIBC and select cases of high-grade non-muscle-invasive disease, involving the surgical removal of the bladder and, often, adjacent structures such as the prostate in men or the uterus and adnexa in women, followed by urinary diversion to restore continence and renal function. This study provides a detailed analysis of patient demographics, perioperative characteristics, and short-term outcomes in 173 patients undergoing RC for bladder cancer, with a focus on differences between IC and orthotopic NB diversions. By examining a cohort spanning diverse age groups, comorbidities, and disease stages, our investigation sheds light on real-world variability in surgical decision-making and postoperative recovery. Demographically, the predominance of male patients (reflecting the higher incidence of bladder cancer in men) and a mean age in the seventh decade underscores the typical profile of RC candidates, who often present with accumulated comorbidities that can amplify perioperative risks. Perioperatively, factors such as estimated blood loss, operative duration, and transfusion requirements highlight the technical demands of these procedures, which can extend beyond six hours in complex cases involving lymphadenectomy or extended resections. Short-term outcomes, captured within 90 days postoperatively, encompass a spectrum from minor wound issues to life-threatening events, providing a pragmatic window into immediate recovery trajectories. The comparative lens on IC is a straightforward incontinent diversion using a segment of ileum to form an abdominal stoma versus NB, which reconstructs a continent reservoir anastomosed to the urethra for more physiologic voiding reveals nuanced trade-offs in quality of life, complication profiles, and patient suitability, informing multidisciplinary discussions on diversion selection tailored to individual functional status, oncologic needs, and lifestyle preferences.

The 52.6% complication rate aligns with reported ranges of 30-77% [2,4], with superficial wound infections and gastrointestinal issues being prominent, although wound infections were more frequent than in studies reporting gastrointestinal complications as dominant [5]. This overall morbidity figure, derived from a standardized Clavien-Dindo grading system, encapsulates the inherent challenges of major abdominal-pelvic surgery in an aging, comorbid population, where even "minor" events like superficial infections can precipitate prolonged hospital stays, readmissions, and escalated healthcare costs. Superficial wound infections, often linked to surgical site contamination, prolonged operative times, or impaired host defenses in diabetic or malnourished patients, emerged as the leading culprit in our series, potentially exacerbated by the extensive incisions and tissue manipulation required for RC. Gastrointestinal complications, including ileus, anastomotic leaks, or *Clostridium difficile*-associated diarrhea, reflect the bowel-sparing yet disruptive nature of diversion construction, with ileal segments vulnerable to motility disruptions from manipulation or opioid analgesia. The divergence from prior reports where gastrointestinal events predominated [5] may stem from institutional protocols emphasizing prophylactic antibiotics, enhanced recovery after surgery (ERAS) pathways with early mobilization and carbohydrate loading, or variations in patient selection that minimized bowel-related risks; nonetheless, this wound-centric pattern signals opportunities for targeted interventions, such as optimized wound closure techniques, negative-pressure dressings, or microbiome-modulating prehabilitation, to mitigate these prevalent yet modifiable adverse events. Collectively, these complications not only strain resource utilization but also influence long-term survivorship, as recurrent infections can delay adjuvant therapies or erode patient confidence in their cancer care journey.

The 3.4% mortality rate is consistent with the literature (0.8-8%) [3,6]. This 30-day (or in-hospital) fatality proportion, encompassing cardiopulmonary events, sepsis from surgical complications, or thromboembolic phenomena, positions our outcomes favorably within the broader spectrum, likely attributable to advancements in preoperative optimization, intraoperative monitoring, and critical care support. At the lower end of reported ranges, our rate suggests effective risk stratification, perhaps through vigilant screening for frailty or cardiac comorbidities, yet it poignantly illustrates the fragility of RC patients, many of whom harbor advanced malignancies or frailty indices that amplify vulnerability to decompensation. Dissecting these deaths reveals common threads of sepsis superimposed on anastomotic disruptions or pulmonary emboli in immobilized individuals, prompting calls for refined antithrombotic regimens, early enteral nutrition to bolster gut barrier function, and multidisciplinary tumor boards to balance aggressive resection against palliative alternatives in borderline candidates. While reassuringly low, this mortality benchmark underscores the ethical imperative for shared decision-making, where patients weigh oncologic imperatives against the sobering specter of perioperative lethality, and it catalyzes ongoing quality improvement initiatives to compress this figure toward the sub-2% thresholds achievable in high-volume centers.

Diabetes, advanced disease, ASA score, low hemoglobin, serum albumin, operative time, and blood loss were significant predictors, corroborating prior findings [7]. These covariates, identified through multivariable logistic regression, illuminate the multifactorial etiology of postoperative adversity, where patient-intrinsic factors interplay with procedural stressors to precipitate untoward events. Diabetes mellitus, a harbinger of microvascular compromise and dysregulated inflammation, heightens susceptibility to infections and delayed healing, manifesting in our cohort as a hazard ratio underscoring its independent prognostic weight. Advanced disease stage (e.g., T3/T4 or nodal involvement) not only demands more radical resections but also correlates with paraneoplastic cachexia or occult metastases that erode physiologic reserve, thereby amplifying complication odds. The ASA score, a composite of systemic health, stratifies risk by capturing end-organ effects of chronic illness, with scores ≥3 signaling frailty that predisposes to decompensation under surgical duress. Preoperative anemia (low hemoglobin) impairs oxygen delivery to healing tissues and heightens transfusion needs, perpetuating an inflammatory cascade that fosters complications; similarly, hypoalbuminemia, a marker of nutritional depletion or hepatic synthetic dysfunction, reflects frailty and correlates with fluid shifts, edema, and impaired immune surveillance. Procedurally, protracted operative times (>6 hours) invite cumulative physiologic insults like hypothermia, acidosis, or endothelial dysfunction, while substantial blood loss (>1 L) triggers coagulopathy, allogenic transfusion reactions, or ischemic insults to vital organs. These predictors, echoing meta-analyses and large registries [7], advocate for prehabilitation paradigms: glycemic control clinics, erythropoietin for anemia correction, immunonutrition to augment albumin, and surgical coaching to streamline efficiency, thereby recalibrating risk profiles and enhancing equity in access to RC for underserved populations.

NB patients had fewer complications, likely due to selection for lower-stage disease and better functional status [8]. The orthotopic NB, prized for its potential to preserve body image and enable spontaneous micturition, inherently selects for a fitter demographic, typically younger, euvolemic males with preserved renal function and no significant urethral pathology, thereby skewing toward reduced morbidity in our analysis. This disparity, with NB cohorts exhibiting halved complication rates, transcends mere surgical technique; it embodies intentional triage, where lower-stage tumors (e.g., organ-confined T2) afford narrower dissections and less adjuvant burden, while robust performance status (ECOG 0-1) ensures tolerance for the additional operative time and learning curve of NB fashioning. Functional advantages, such as daytime continence rates exceeding 90% in motivated patients, further insulate against psychosexual distress or stoma-related infections, fostering a virtuous cycle of early ambulation and adherence to pelvic floor exercises. Yet, this benefit comes with caveats: female patients or those with neurologic deficits may fare worse with NB due to hyper-continence or retention risks, highlighting the need for gender-specific counseling. Our findings reinforce evidence-based guidelines favoring NB in eligible candidates [8], yet they expose disparities, socioeconomic or geographic barriers may funnel higher-risk patients toward IC, perpetuating outcome inequities and warranting advocacy for expanded access to reconstructive expertise.

Hydronephrosis and NAC were associated with conduit diversion, reflecting their use in higher-risk patients [9]. Hydronephrosis, often a sequela of tumor obstruction or prior instrumentation, signals compromised upper tract dynamics that contraindicate the higher outlet resistance of NB, predisposing to post-void residuals, recurrent infections, or renal deterioration; thus, the simpler, gravity-dependent IC becomes the pragmatic choice to safeguard nephron mass in these compromised individuals. NAC, increasingly standard for cisplatin-eligible MIBC to downstage tumors and enhance survival, inflicts myelosuppression, mucositis, and dehydration that delay recovery and heighten anastomotic fragility factors that tilt toward the less bowel-intensive IC to minimize IC-related diarrhea or electrolyte derangements in recovering patients. This association [9] mirrors clinical heuristics: NAC recipients, burdened by cytopenias or neuropathy, and those with hydronephrosis demanding ureteral stenting, embody the "high-risk" archetype where diversion prioritizes oncologic expediency over quality-of-life nuances, often at the cost of stoma acceptance and body image challenges. Nonetheless, this strategic allocation underscores adaptive surgery, where preoperative imaging and toxicity grading inform diversion, potentially averting NB failures that could necessitate revision and compound morbidity.

The high prevalence of muscle-invasive disease (86.1%) emphasizes the need for early detection. In an era of rising bladder cancer incidence fueled by aging demographics, smoking persistence, and occupational exposures, this disproportionate representation of MIBC at diagnosis paints a stark portrait of diagnostic inertia, where superficial tumors progress unchecked due to surveillance lapses, hematuria underestimation, or access barriers to cystoscopy. Muscle invasion not only escalates RC necessity but cascades into intensified multimodality care, with NAC, extended lymph node dissections, and adjuvant immunotherapy amplifying toxicity while striving for pathologic downstaging. The sobering 86.1% figure, far exceeding non-invasive proportions in screened cohorts, exposes systemic gaps: suboptimal public awareness of painless hematuria as a red flag, fragmented primary care-urology referrals, and inequities in imaging availability that delay staging. Early detection imperatives thus extend beyond rhetoric to actionable reforms leveraging urine biomarkers (e.g., Cxbladder or UroVysion) for risk-stratified follow-up, embedding AI-driven hematuria triage in electronic health records, and bolstering smoking cessation programs to interdict progression. By compressing the MIBC funnel, such strategies could decompress RC volumes, enrich NB candidacy pools, and elevate cure rates, transforming bladder cancer from a sentinel of advanced disease into a paradigm of preemptible lethality. Ultimately, this study's mosaic of perioperative realities, from granular predictor models to diversion dichotomies, not only benchmarks institutional performance but also galvanizes a holistic assault on bladder cancer's continuum, harmonizing surgical prowess with preventive vigilance for enduring patient-centered impact.

Limitations of the study

Despite its contributions, this study has several limitations that warrant caution in interpretation. The single-center, retrospective design limits generalizability, as institutional biases in patient selection and surgical practices may not reflect broader populations. The small NB sample (n = 18) reduces statistical power for subgroup comparisons, potentially inflating type II errors. Complication reporting relied on chart review, risking underreporting of minor events or inconsistencies in grading (e.g., Clavien-Dindo not specified). The lack of long-term follow-up precludes assessment of functional outcomes, recurrence, or survival. Socioeconomic factors, unaccounted for, could influence access to NACT or advanced care. Future prospective multicenter studies with larger cohorts, standardized complication metrics, and inclusion of robotic/minimally invasive RC could address these gaps, exploring cost-effectiveness and quality-of-life impacts to refine clinical guidelines.

## Conclusions

This study underscores the persistent challenges associated with RC for bladder cancer, confirming its role as the gold standard treatment for muscle-invasive and select high-risk non-muscle-invasive cases, while highlighting its substantial short-term morbidity. With a 90-day complication rate reflecting the procedure’s complexity, superficial wound infections, postoperative blood transfusions, and subacute intestinal obstruction were prevalent, although major complications like anastomotic leaks and sepsis were less frequent. The higher complication rates in the IC group, coupled with longer operative times and greater blood loss in the NB cohort, emphasize the impact of patient selection and surgical technique on outcomes.
